# Overexpression of *NtSOS2* From Halophyte Plant *N. tangutorum* Enhances Tolerance to Salt Stress in *Arabidopsis*

**DOI:** 10.3389/fpls.2021.716855

**Published:** 2021-09-06

**Authors:** Liming Zhu, Mengjuan Li, Junnan Huo, Ziming Lian, Yuxin Liu, Lu Lu, Ye Lu, Zhaodong Hao, Jisen Shi, Tielong Cheng, Jinhui Chen

**Affiliations:** ^1^Key Laboratory of Forest Genetics and Biotechnology of Ministry of Education of China, Co-Innovation Center for Sustainable Forestry in Southern China, Nanjing Forestry University, Nanjing, China; ^2^College of Biology and the Environment, Nanjing Forestry University, Nanjing, China

**Keywords:** salt stress, *SOS2*, salt overly sensitive pathway, *Nitraria tangutorum*, halophyte plant

## Abstract

The Salt Overly Sensitive (SOS) signaling pathway is key in responding to salt stress in plants. *SOS2*, a central factor in this pathway, has been studied in non-halophytes such as *Arabidopsis* and rice, but has so far not been reported in the halophyte *Nitraria tangutorum*. In order to better understand how *Nitraria tangutorum* acquires its tolerance for a high salt environment, here, the *NtSOS2* was cloned from *Nitraria tangutorum*, phylogenetic analyses showed that NtSOS2 is homologous to the SOS2 of *Arabidopsis* and rice. Gene expression profile analysis showed that *NtSOS2* localizes to the cytoplasm and cell membrane and it can be induced by salt stress. Transgenesis experiments showed that exogenous expression of *NtSOS2* reduces leaf mortality and improves the germination rate, biomass and root growth of *Arabidopsis* under salt stress. Also, exogenous expression of *NtSOS2* affected the expression of ion transporter-related genes and can rescue the phenotype of *sos2-1* under salt stress. All these results revealed that *NtSOS2* plays an important role in plant salt stress tolerance. Our findings will be of great significance to further understand the mechanism of salt tolerance and to develop and utilize molecular knowledge gained from halophytes to improve the ecological environment.

## Introduction

Soil salinization has become a major constraint to the sustainable development of the ecological environment, more than 20% of arable land suffered from soil salinization (Shrivastava and Kumar, 2015; Rui and Ricardo, 2017) and a trend for ever increasing salinization has been observed (Cuevas et al., 2019; Singh, 2021). The area of salinized soil in the world is gradually expanding due to human activity and natural phenomena. Soil salinization seriously inhibits plant growth (Tian et al., 2020) through soil hardening and decreases in soil total porosity and fertility, in general lowering a plant’s chances of survival (Jez et al., 2016; Van Zelm et al., 2020). Excessively high salt concentrations make it difficult for plants to germinate, causing plant biomass to decline or even to die, hindering the comprehensive utilization and sustainable development of soil.

The main reason why plants have difficulty in living in salinized soil is that they are subjected to abiotic stress, which will lead to osmotic stress and ion toxicity under salt stress (Liang et al., 2018; Arif et al., 2020). When plants subjected to stress, Ca^2+^ concentration increases and activates protein kinases by calmodulin then induce a series of cellular reactions to make plants adapt to or reduce the damage caused by salt stress (Villalobo and Berchtold, 2020). Plant protein kinases (a kind of phosphotransferase) participate in the process of plant stress resistance signaling and play an important role when plants suffer from salt damage and drought stress (Yip Delormel and Boudsocq, 2019; Chen et al., 2021). Calcineurin B-like proteins-interacting protein kinases (CIPKs) are plant-specific protein kinases, which contain conserved Protein Kinase and NAF/FLSL domains (Tang et al., 2020). CIPKs are involved in a variety of abiotic stresses such as salt stress and drought stress, and have been identified and functionally validated in plant (Ma et al., 2019, 2020). *CIPK24* is also known as *SOS2* in *Arabidopsi*s and encodes a serine/threonine protein kinase with two important functional domains: an N-terminal catalytic domain and a C-terminal regulatory domain. *SOS2* was first identified and cloned from *Arabidopsis* using bitmap cloning methods and was found to play an important role in the SOS signaling pathway, which maintaining the ion balance within plant cells (Liu et al., 2000). The *SOS2* C-terminal regulatory region contains a conserved NAF/FISL motif that interacts with the Ca^2+^ binding protein *SOS3*/*CBL4* to form a binary complex, after which they integrate into the plasma membrane and activate the *SOS1* gene (Quintero et al., 2002; Qiu et al., 2004; Yang et al., 2009).

*Nitraria tangutorum* is a species within the Nitraria Linnaeus genus, which is widely distributed in arid saline-alkali areas within northwest China (Zhou et al., 2015). *Nitraria tangutorum* has a strongly developed root system which can withstand sand burial, and has excellent tolerance to drought and salinity. It is one of the outstanding pioneer species in a saline-alkali environment, making it an excellent model species for research into salt/drought stress resistance (Liu et al., 2016). So, the use of *Nitraria tangutorum* to reduce the accumulation of salt on the soil surface and can repair saline-alkali land up to a point. At present, research on *Nitraria tangutorum* has mainly focused on the determination of physiological and biochemical indices of stress resistance and fruit composition (Liu et al., 2014), with research into the molecular mechanism of salt tolerance being quite scarce.

Furthermore, *SOS2* has been primarily studied more in well-known plant model species such as *Arabidopsis* and rice (Batelli et al., 2007; Martínez-Atienza et al., 2007; Fujii and Zhu, 2009), with such studies indicating that *SOS2* plays a key role in plant saline-alkali resistance. Summarizing, few molecular studies on *Nitraria tangutorum*, such as on the *NtCIPK2* and *NtCIPK9* genes, have been reported (Zheng et al., 2014; Lu et al., 2020), while any study on the *Nitraria tangutorum SOS2* gene is still lacking.

Salt tolerant plants can grow in salinized soil. Therefore, it is a great significance to understand the mechanism of salt tolerance in halophytes in order to alleviate and improve soil salinization. Studies on model species have shown that *SOS2* plays an important role in the process of plant stress tolerance, so a key question is how does this gene respond to salt stress in the halophyte *Nitraria tangutorum*? To explore the role of *SOS2* in *Nitraria tangutorum*, the *NtSOS2* gene was cloned and analyzed its expression pattern under salt stress. Meanwhile, to explore whether the exogenous expression of *NtSOS2* may affect Arabidopsis salt resistance, the germination rate, leaf white blade rate, fresh weight, root length and phenotype of transgenic Arabidopsis were analyzed and compared it to the wild type. Our results indicated that exogenous expression of *NtSOS2* enhances Arabidopsis salt tolerance. These experiments indicated that *NtSOS2* plays a key role in plant salinity tolerance.

## Materials and Methods

### Plant Materials and Growth Conditions

*Nitraria tangutorum* seeds were collected from Dengkou county, Inner Mongolia, China. The seeds were mixed with wet sand at 4°C for ∼2 months to vernalize, then placed on germination box. After germination, seedlings were planted in a matrix with a peat soil: perlite ratio of 4:1 and grown at 23°C with a 16-h light/8-h dark light cycle and 60% air humidity. Seedlings were grown for 3 months, after which they were treated with a 500 mM NaCl solution, collected at 0, 1, 6, 12, and 24 h intervals, then submerged in liquid nitrogen to quickly freeze for subsequent RNA extraction.

The *Arabidopsis thaliana* Columbia ecotype (Col-0) used in this study were provided by Prof. Thomas Laux (Signalling Research Centres BIOSS and CIBSS, Faculty of Biology, University of Freiburg, Germany); The mutant *sos2-1* obtained from Nottingham Arabidopsis Stock Centre (NASC ID: N3863). These seeds were sown on 1/2 MS medium after disinfection, stored at 4°C for 3 days to stratify, after which they were cultivated in a light incubator having identical culture conditions as *Nitraria tangutorum* above. When seedlings reached the four leaves stage, they were transferred to a substrate of peat soil: vermiculite: perlite with 5:1:1 ratio for further cultivation.

### Cloning and Sequence Analysis of *NtSOS2*

Total ribonucleic acid was isolated from *Nitraria tangutorum* leaves with an RNA extraction kit (Bioteke, China) and treated with DNase I (Takara, Japan) to remove contaminating desoxyribonucleic acid. cDNA was synthesized with 1st strand cDNA synthesis Kit (Vazyme, China). The *NtSOS2* was amplified from *Nitraria tangutorum*’s cDNA using specific primers which designed by Oligo 7.60 (Cascade, CO 80809, United States) that were listed in Supplementary Table 1. Homologous proteins were searched for using the NCBI blastp program and multiple alignments were performed using DNAMAN v9.0 (Lynnon Corporation, San Ramon, CA, United States) with the deduced amino acid sequences. A phylogenetic tree was constructed through the Neighbor-Joining method, and the bootstrap value was calculated according to 1,000 repetitions using MEGA-X v10.1 (Temple, Philadelphia, PA, United States).

### Quantitative Real-Time PCR Analyses

Quantitative real-time PCR (qRT-PCR) was performed using the AceQ qPCR SYBR Green Master Mix (Vazyme, China). Cycling conditions were 94°C for 15 s, 60°C for 30 s, and 72°C for 30 s. Each reaction was performed in triplicate. Relative expression values were calculated through the relative quantization method (2^–ΔΔ*CT*^) (Livak and Schmittgen, 2001), primer and actin sequences for qRT-PCR are listed in Supplementary Table 2.

### Subcellular Localization

The *NtSOS2* open reading frame (ORF) was fused in-frame upstream to GFP in pJIT166 vector using *Xba*I and *Bam*HI restriction sites. The primers used to construct this vector were listed in Supplementary Table 3. Then the constructive fusion plasmid was transfected into onion epidermal cells by microprojectile bombardment (Nebenführ, 2014) and incubated for 24 h in the dark. The cells were subsequently observed using Zeiss fluorescence microscope (X-cite 120Q, Carl Zeiss).

### *Arabidopsis* Transformation

A pair of primers (listed in Supplementary Table 4) was designed to amplify the ORF of *NtSOS2* and clone it into the pBI121 vector using *Xba*I and *Bam*HI restriction sites. The vector was then transformed into *Arabidopsis columbia* and *sos2-1* mutant using the method reported by Clough and Bent (1998), after which positive plants were screened using kana-containing 1/2MS medium. Transformed plants were confirmed using PCR amplification.

### Analysis of Salt Tolerance of Transgenic Arabidopsis

To analyze the germination rate, transgenic Arabidopsis of the T3 generation and Col-0 were seeded on 9 × 9 cm square dishes containing 1/2 MS medium supplemented with 0, 100, or 150 mM NaCl. ∼70 seeds were placed on each dish for each strain, plants were then cultured for 7 days in the light incubator, after which the germination rate was determined. For phenotypical observation (root length, leaf blade whitening ratio and fresh weight), seedlings were grown on 1/2 MS culture medium containing 0, 100, or 150 mM NaCl for 10 days of vertical culture. They were then transferred to soil for another 2 weeks of growth, after which 200 mM NaCl was used to induce salt stress and surface appearance and leaf mortality rate were scored. For rescue phenotype of *sos2-1*, the root length and fresh weight are observated seedlings were grown on 1/2 MS culture medium containing 0, 60, or 120 mM NaCl for 7 days of vertical culture and then transferred to soil culture are the same as above conditions, after which 150 mM NaCl was used to induce salt stress and surface appearance and leaf mortality rate were scored. Each experiment was performed using three biological replicates.

### Analyze Physiological Changes of Salt Stress

The *Arabidopsis* growing in soil were exposed to salt solution for 3 days for physiological determination. The Soluble protein, Superoxide Dismutase (SOD), Malondialdehyde (MDA) were determinated and analyzed by the soluble protein, superoxide dismutase and malondialdehyde assay kits, respectively (Jiancheng Bioengineering Institute, Nanjing, China).

For the determination of chlorophyll, the protocol referred to the previously reported method (Lichtenthaler and Wellburn, 1983), 0.1 g fresh leaves were taken and cut into 10 ml 96% ethanol. The absorbance was measured at 665 and 649 nm under dark conditions at room temperature until the leaves turned white completely. Each experiment was performed using three biological replicates.

## Results

### Cloning and Sequence Analysis of *NtSOS2*

In order to clone *SOS2* of *Nitraria tangutorum*, the SOS2 protein sequences from rice and *Arabidopsis* were compared to our unpublished transcriptome data using the NCBI blastp program. A pair of specific primers were designed to obtain the SOS2 target sequence by PCR amplification (Supplementary Figure 1). Gene sequence analysis showed that *NtSOS2* coding sequence (CDS) length is 1,332 bp which encodes a protein 443 amino acids in length, with molecular weight of 50.34 kDa.

**FIGURE 1:**
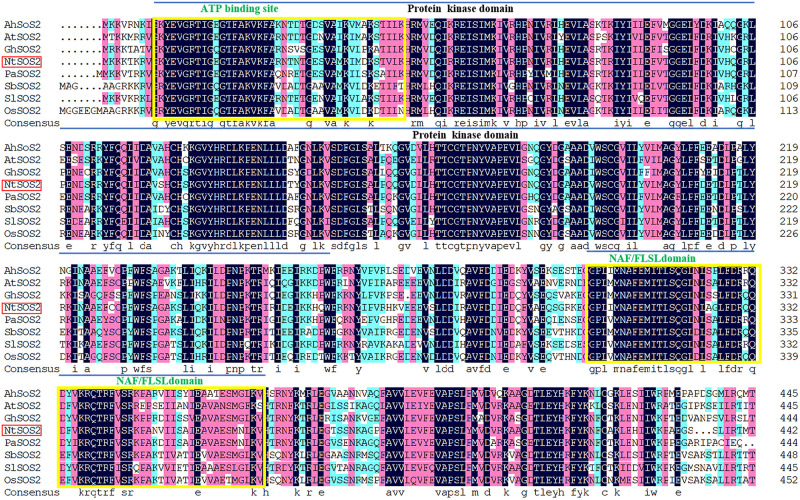
Multiple amino acid sequence alignment of NtSOS2. The amino acid from *Arachis hypogaea* (HG797656.1), *Arabidopsis thaliana* (NM_122932.5), *Gossypium hirsutum* (GU_188961.1), *Populus alba* (KT_875331.1), *Sorghum bicolor* (KY_202762.1), *Solanum lycopersicum* (NM_001247281.2), *Oryza sativa* (XP_015643084.1), the blue lines represent the Protein Kinase and NAF/FLSL domain, respectively.

Then DNAMAN software was used to compare the NtSOS2 protein sequence to homologous sequences from seven additional plant species and found the amino acid sequence homology of NtSOS2 to the other seven species to be average of 82.01% (Figure 1), indicating that NtSOS2 has a relatively high sequence similarity to homologs from other species. In addition, the NCBI conserved domain program was used to identify conserved Protein Kinase (PF00069) and NAF/FLSL (PF03822) domains (Supplementary Figure 2), showing that *NtSOS2* is a typical CIPK gene as these domains are essential for CIPK function.

**FIGURE 2:**
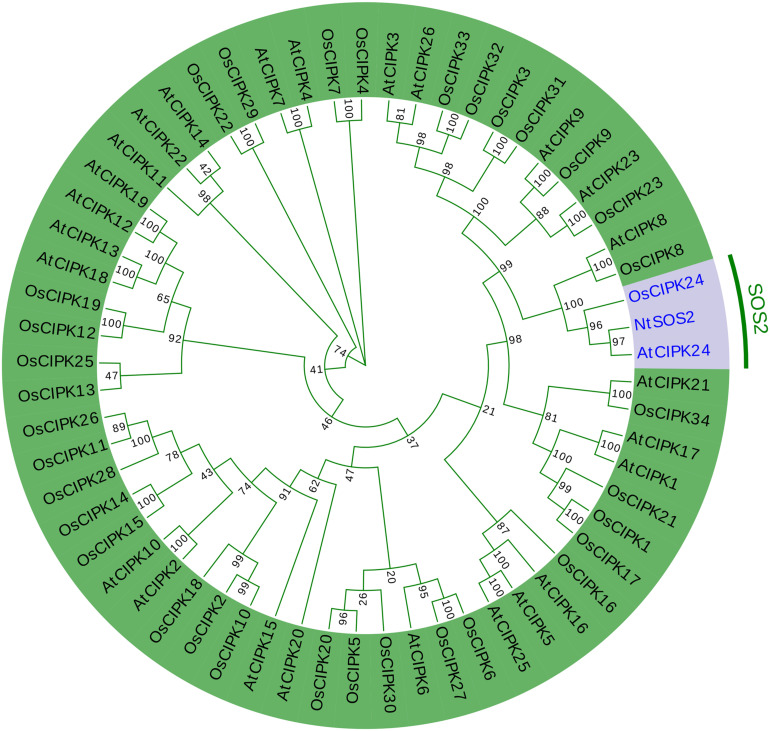
Phylogenetic analysis of NtSOS2 with Arabidopsis CIPKs and Rice CIPKs. The red section highlights the SOS2 gene of all three species, while the green section represents all other *CIPK* gene family members from *Arabidopsis* and rice.

Furthermore, the NtSOS2 protein sequence was aligned with the CIPK gene family of *Arabidopsis* and rice to construct a phylogenetic tree, and the results showed that NtSOS2, AtCIPK24, and OsCIPK24 are distributed on one branch (Figure 2). This showed that *NtSOS2* has a high homology to *AtCIPK24* and *OsCIPK24* that have been previously described as SOS2 genes, which indicated that NtSOS2 may have similar functions compared with SOS2 of *Arabidopsis* and rice.

### Subcellular Localization of NtSOS2

**FIGURE 3:**
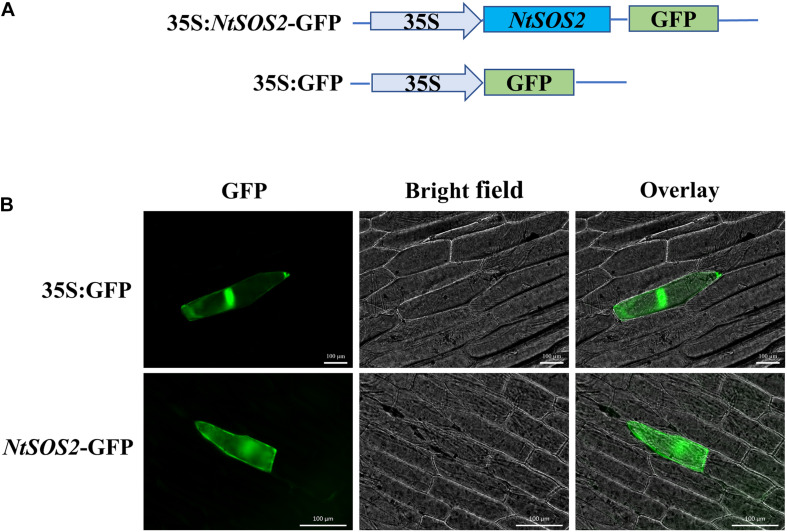
Subcellular localization of NtSOS2-GFP. **(A)** Schematic diagram of the SOS2 subcellular localization and GFP control vectors. **(B)** Fluorescence microscopy analysis of the GFP signal from onion epidermal cells transiently expressing GFP and NtSOS2-GFP fusions as indicated. Scale bar = 100 μm.

To understand the expression pattern of *NtSOS2*, the ProtComp 9.0 tools was used to predict and analyze the subcellular localization of NtSOS2, and the result showed that the probability of NtSOS2 localizing to the cytoplasm and cell membrane is relatively high (Supplementary Figure 1). To further determine the subcellular localization of NtSOS2, the coding region of NtSOS2 was combined into a pJIT166-GFP vector. Then, this plasmid was instantly expressed in onion epidermal cells via microparticle bombardment and 35S:GFP was used as a control. As shown in Figure 3, 35S:GFP could be detected in the nucleus and cytosol, while 35S:NtSOS2-GFP was detected in the cytoplasm and plasma membrane, consistent with the subcellular localization prediction of ProtComp.

### Expression Analysis of *NtSOS2* Under Salt Stress

**FIGURE 4:**
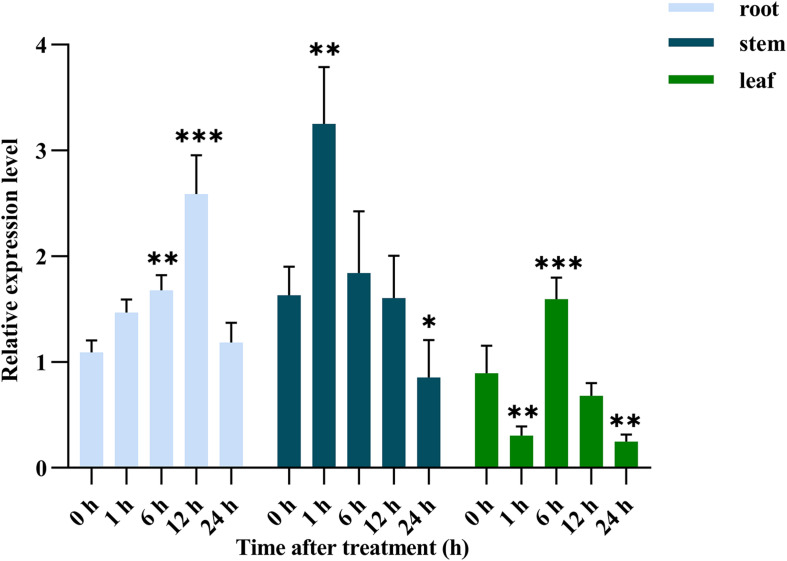
Relative expression of *NtSOS2* in roots, stems and leaves under 500 mM NaCl salt stress. Displayed is the mean ± SD. Error bars indicate the standard deviation of three biological replicates. Statistically significant differences were assessed using ANOVA test (^∗^*P* < 0.05, ^∗∗^*P* < 0.01, ^∗∗∗^*P* < 0.001). The significant differences were analyzed in root, stem and leaf, respectively transgenic lines.

Abiotic stress can induce changes in the expression of SOS2 (Kaur et al., 2015), the *Nitraria tangutorum* seedlings were stressed in solution containing 500 mM NaCl to investigate whether SOS2 had a similar expression pattern, the root, stem and leaf tissue were collected at 0, 1, 6, 12, and 24 h, respectively, for real-time quantitative PCR analysis of *NtSOS2* expression. *NtSOS2* expression in the root continuously increased in response to 500 mM NaCl between 0 and 12 h, followed by downregulation at 24 h, while *NtSOS2* expression in the stem reached its maximum 1 h after stress exposure (Figure 4). In the leaf, the expression of *NtSOS2* was observed a more wave-like pattern, with an expression peak observed after 6 h. Overall, *NtSOS2* expression under NaCl stress tended to be up-regulated first, then down-regulated.

### Exogenous Expression of *NtSOS2* Enhances Germination Under Salt Stress in *Arabidopsis*

In order to verify the potential function of *NtSOS2*, an overexpression vector of *NtSOS2* was constructed and transformed into *Arabidopsis*, three of transgenic lines with relatively high expression of *NtSOS2* were selected through semi-quantitative experiments (Supplementary Figure 3).

**FIGURE 5:**
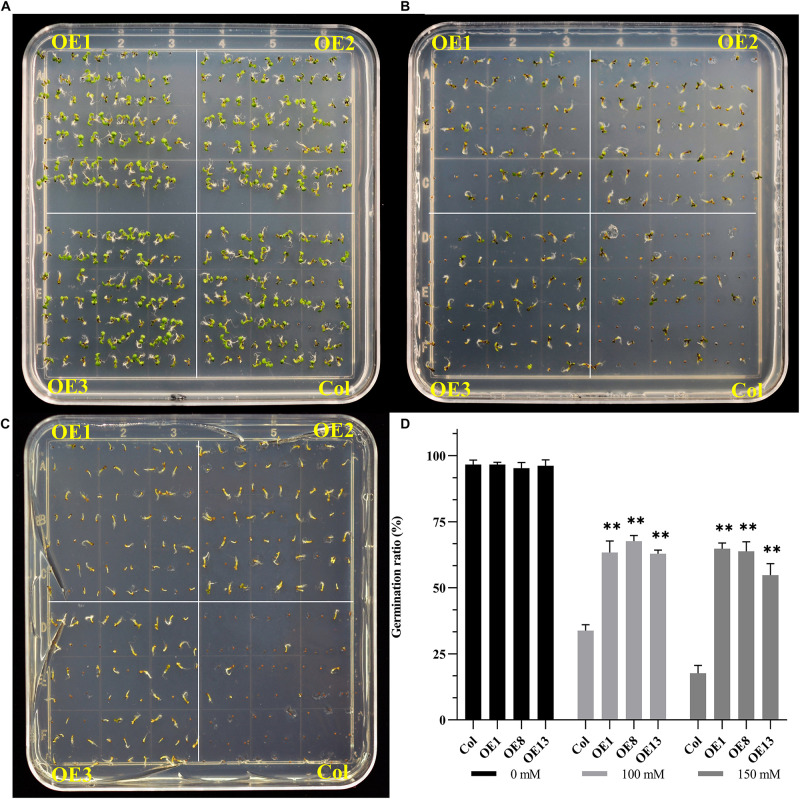
Exogenous expression of *NtSOS2* increases germination rate under salt stress. **(A–C)** Wild-type (Col) and transgenic (OE1-3) Arabidopsis seeds germinating on 1/2 MS medium supplemented with NaCl (0, 100 mM, 150 mM) 7 day after being transferred to the light. **(D)** Quantification of germinate rate. *n* = 70 seeds for each condition (genotype/NaCl conc.). The germination rate is shown as the mean ± SD (*n* = 3). Statistically significant differences were assessed using an ANOVA test (^∗∗^*P* < 0.01). OE1-3 represent three independent transgenic lines.

Salt stress can significantly inhibit the germination of seeds (Zhang et al., 2019). Since *NtSOS2* expression increases in response to salt stress, whether exogenous *NtSOS2* expression may increase germination rate under salt stress conditions, so wild type and transgenic Arabidopsis over expressing *NtSOS2* were grown on 1/2 MS medium containing 0, 100, and 150 mM NaCl for 7 days. The result showed that the germination rate of transgenic Arabidopsis was not affected under conditions without salt stress (Figures 5A,D). However, in the 1/2MS medium containing 100 mM NaCl, the germination rate of wild-type seeds decreased significantly, which may be due to the significant inhibition of *NtSOS2* over expression (Figures 5B,D).

The decline in germination rate of wild type seeds was even more pronounced when applying 150 mM salt stress, while germination of transgenic seeds did not decrease further (Figures 5C,D). In wild type Arabidopsis the germination rate decreased from 96.66 to 17.14% under heavy salt stress on average, in transgenic seeds the germination rate decreased to 62.38% on average under heavy salt stress, a much less dramatic effect. Therefore, these data demonstrate that overexpression of *NtSOS2* can significantly decrease the effect that salt stress has on seed germination rate in *Arabidopsis*.

### Exogenous Expression of *NtSOS2* Enhances Salt Tolerance in *Arabidopsis*

**FIGURE 6:**
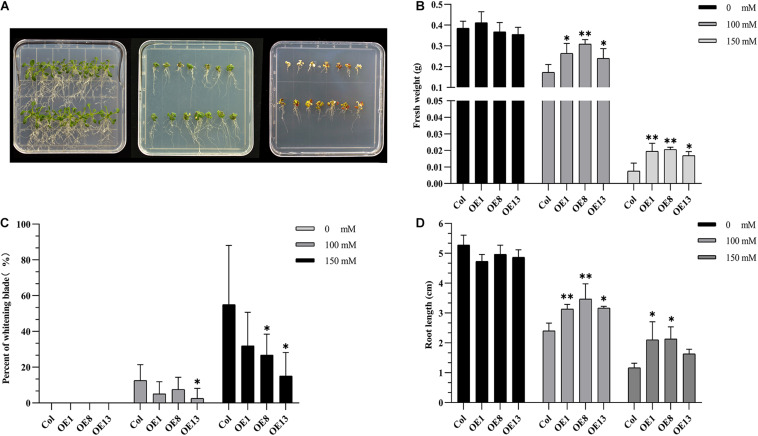
Exogenous expression of *NtSOS2* enhances salt tolerance in Arabidopsis. **(A)** Wild-type (top-rows) and *NtSOS2* overexpressing (bottom rows) Arabidopsis were grown on 1/2 MS medium containing 0 (left), 100 (middle) and 150 mM (right) NaCl for 10 days. **(B–D)** Fresh weight (g) **(B)**, Leaf blade whitening (%) **(C)** and Main root length (cm) **(D)** of wild-type and *NtSOS2* overexpressing Arabidopsis under salt stress. See legend for NaCl concentrations used. Each experiment was performed with three biological replicates. An ANOVA test was used for statistical analysis. ^∗^*P* < 0.05, ^∗∗^*P* < 0.01. Scale bar = 1 cm.

**FIGURE 7:**
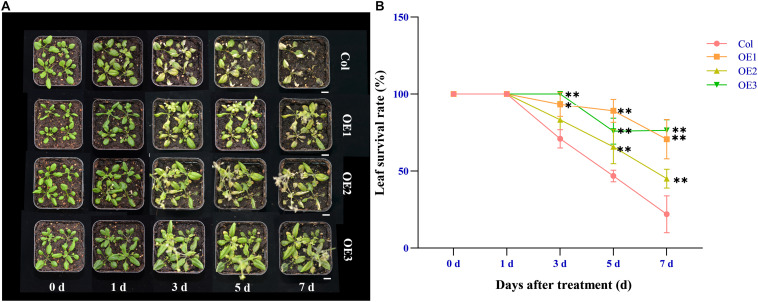
Ectopic expression of *NtSOS2* reduces the leaf mortality rate. **(A)** Representative photograph of wild-type and *NtSOS2* overexpressing lines under 200 mM NaCl stress. **(B)** The leaf survival rate under salt stress of wild-type (red) and *NtSOS2* overexpression (orange, green, blue) lines. ANOVA test was used for statistical analysis, ^∗^*P* < 0.05, ^∗∗^*P* < 0.01. Scale bar = 1 cm.

To verify whether *NtSOS2* overexpression can improve the tolerance of Arabidopsis seedlings to salt stress, wild type and transgenic Arabidopsis were grown on vertically placed plates containing 1/2 MS medium supplemented with 0, 100, and 150 mM NaCl for 10 days. On medium without added NaCl, wild type and transgenic Arabidopsis showed no obvious differences in growth, while on 100 or 150 mM NaCl, growth of wild type Arabidopsis was clearly reduced, with wild type plants growing on 150 mM NaCl clearly showing stronger whitening and anthocyanin accumulation than *NtSOS2* overexpressing plants (Figure 6A). In addition, *NtSOS2* overexpression significantly reduces loss of fresh weight, leaf whitening and inhibition of root growth under high salt stress conditions (Figures 6B–D). To continue observing plant growth past the seed stage, four-leaved seedlings were transferred to soil, growing them for 2 weeks with 200 mM NaCl irrigation. After 1 day of growth, no differences could be observed, but starting from 3 days of growth, wild-type Arabidopsis showed significantly more leaf mortality than *NtSOS2* overexpressing lines (Figures 7A,B).

**FIGURE 8:**
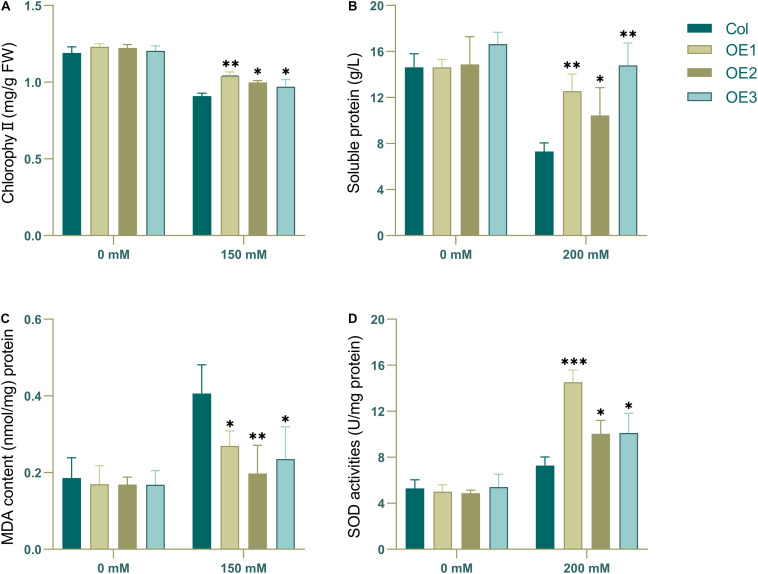
The chlorophyll, MDA and antioxidative enzyme activity analyses. 2-week-old wild type and transgenic *Arabidopsis* were treated with 0 or 200 mM NaCl for 3 days. **(A)** Chlorophyll content. **(B)** Soluble protein content. **(C)** MDA level. **(D)** SOD activity. ANOVA test was used for statistical analysis. ^∗^*P* < 0.05, ^∗∗^*P* < 0.01, ^∗∗∗^*P* < 0.001.

Judging from the results, there were more obvious phenotypic differences after 3 days of salt stress for wild type and transgenic type. To characterize their change in physiological state, wild-type and transgenic Arabidopsis that had been subjected to salt irrigation for 3 days were chosen to detected the chlorophyll, soluble protein, MDA and SOD content. The results showed that in the absence of salt stress, there was no significant difference in chlorophyll content between transgenic lines and wild-type lines (Figure 8). However, there were significant differences in chlorophyll, soluble protein, MDA and SOD content between transgenic and wild-type strains under salt stress. The content of chlorophyll, soluble protein and SOD in the transgenic plants was higher than that in the wild-type, while the content of MDA was the opposite (Figure 8), which may indicate that the wild-type plants showed higher salt intolerance. Taken together, these results show that *NtSOS2* can counteract the physiological effects that high salt stress has on growth of *Arabidopsis*, thereby endowing plants with increased salt tolerance.

### Exogenous Expression of *NtSOS2* Can Rescue Mutant *sos2-1* Phenotype

**FIGURE 9:**
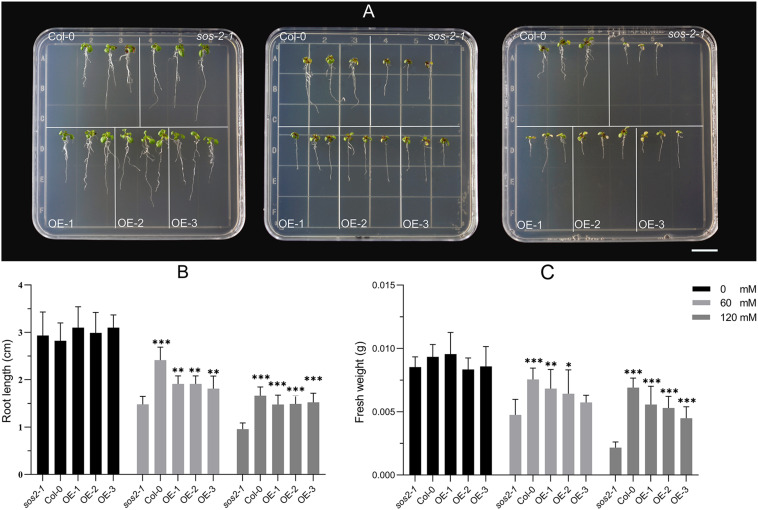
Exogenous expression of *NtSOS2* could rescue *sos2-1* phenotype on salt stress. **(A)** Col, mutant *sos2-1* and transgenic Arabidopsis were treated at 0, 60, 120 mM NaCl for 7 days. **(B)** The root length (cm) of *sos2-1* and transgenic Arabidopsis on salt stress. **(C)** The fresh weight (g) of *sos2-1* and transgenic Arabidopsis on salt stress. Three biological replicates for each experiment. ANOVA test was used for statistical analysis. ^∗^*P* < 0.05, ^∗∗^*P* < 0.01, ^∗∗∗^*P* < 0.01.

In *Arabidposis*, mutates *SOS2* leads to salt stress sensitive phenotype (Liu et al., 2000). Exogenous expression of *CIPK24* in canola and foxtail millet can complement the phenotype of *sos2-1* (Zhang et al., 2014, 2017). In order to verify whether *NtSOS2* has a similar function with other species, the *NtSOS2* gene were exogenously expressed into *sos2-1*. Three of transgenic lines were used for functional analysis (Supplementary Figure 3), on medium without added NaCl, *sos2-1* and transgenic Arabidopsis showed no obvious differences in growth, while on 60 or 120 mM NaCl, growth of mutant *Arabidopsis* was clearly reduced, with *sos2-1* plants growing on 120 mM NaCl clearly showing stronger whitening and anthocyanin accumulation than *NtSOS2* overexpressing plants (Figure 9A).

**FIGURE 10:**
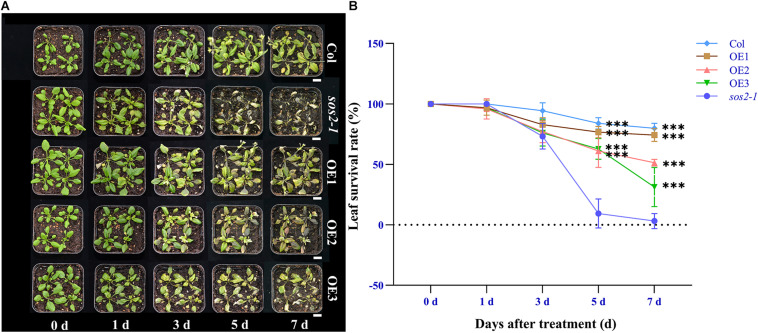
Ectopic expression of *NtSOS2* can rescue *sos2-1* phenotype. **(A)** Phenotype observation of *sos2-1* and overexpression lines under 150 mM NaCl stress. **(B)** Statistics of leaf survival rate under salt stress in *sos2-1*, wild type and overexpression lines. ANOVA test was used for statistical analysis. Scale bar = 1 cm. ANOVA test was used for statistical analysis. ^∗∗∗^*P* < 0.001.

In addition, under high salt stress conditions, the seedings that overexpress the *NtSOS2* gene significantly reduces loss of fresh weight and inhibition of root growth under high salt stress conditions (Figures 9B,C). To continue observing plant growth past the seed stage, four-leaved seedlings were transferred to soil, when growing for 2 weeks with 150 mM NaCl irrigation. After 1 day of growth, no differences could be observed, but starting from 3 days of growth, *sos2-1* showed significantly more leaf mortality than *NtSOS2* overexpressing lines (Figure 10).

**FIGURE 11:**
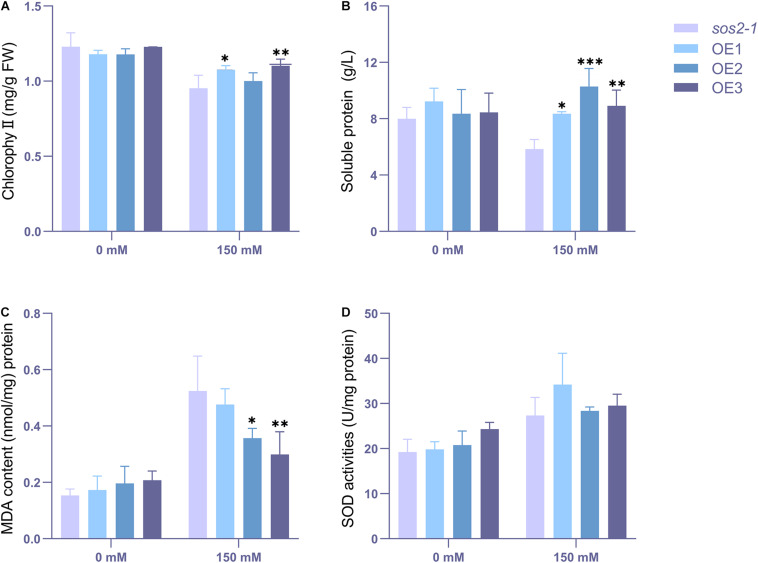
The chlorophyll, MDA and antioxidative enzyme activity analyses. 2-week-old mutant and transgenic *Arabidopsis* were treated with 0 or 150 mM NaCl for 3 days. **(A)** Chlorophyll content. **(B)** Soluble protein content. **(C)** MDA level. **(D)** SOD activity. ANOVA test was used for statistical analysis. ^∗^*P* < 0.05, ^∗∗^*P* < 0.01, ^∗∗∗^*P* < 0.001.

To characterize their change in physiological state, these plants were taken to detected the chlorophyll content, soluble protein, MDA and SOD content (Figure 11), and the results showed that the contents of chlorophyll, soluble protein, MDA and POD were not significantly different between the wild types and transgenic types cultured under normal conditions. However, under salt stress, the contents of chlorophyll, MDA and soluble protein of the mutant were significantly different from those of the transgenic plants. At the same time, the SOD content of the transgenic lines was generally higher than that of the mutant *sos2-1*, although the SOD content did not reach a statistically significant difference (*P*<0.05).

### *NtSOS2* Affects the Expression of Ion Transporter-Related Genes

**FIGURE 12:**
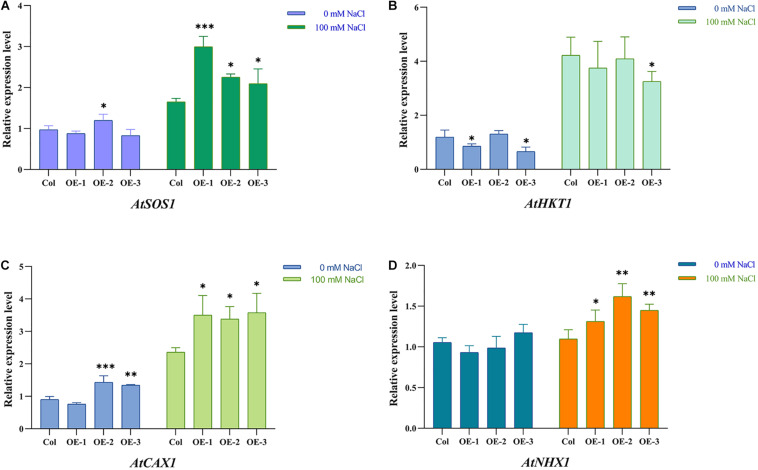
Ectopic expression of *NtSOS2* affected Ion transporter-related genes expression. **(A–D)** Ion transporter-related genes relative quantification. ANOVA test was used for statistical analysis. ^∗^*P* < 0.05, ^∗∗^*P* < 0.01, ^∗∗∗^*P* < 0.001.

In *Arabidopsis*, the *AtSOS2* gene plays an important role in maintaining cellular ion balance. In order to explore whether exogenous expression of *NtSOS2* affects the expression of Arabidopsis ion transport-related genes, 10-day-old wild-type and transgenic Arabidopsis plants were placed on MS medium contains 100 mM NaCl for 7 days of continuous salt stress, after which were collected for fluorescence quantitative PCR experiments. Four genes which reported to be related to ion transport: *AtSOS1*, *AtHKT1*, *AtCAX1*, and *AtNHX1* were selected to detected the change of expression, and the results showed that under salt stress, the expression levels of these genes were significantly increased in both wild-type and transgenic Arabidopsis (Figure 12). Furthermore, the expression of *AtSOS1*, *AtCAX1*, and *AtNHX1* under salt stress was higher in transgenic than in wild-type plants, while the expression level of *AtHKT1* was slightly decreased in transgenic plants under these conditions (Figure 12).

## Discussion

Soil salinization is a common environmental stress factor that causes soil hardening and soil fertility decline, seriously affecting the germination and growth of plants (Litalien and Zeeb, 2020). More and more cultivated land is suffering from saline-alkali harm, making crop yields decrease (Xun et al., 2015) and reducing the grain surplus. At present, there are several known methods to reduce soil saline-alkalization, among which phytoremediation is one of the better methods with a low cost and no side effects to the environment. Plant mulching is beneficial to slow down the evaporation rate of soil water in saline-alkali land and reduces the accumulation of salt in the soil surface layer. Meanwhile, some saline-living plants can also accumulate salt to slow down or treat salinized soil. Therefore, plant remediation has a good prospect for saline-alkali land remediation.

Long-term saline-alkali stress causes halophytes to adapt and change so that they can grow and reproduce in the harsh saline-alkali land. Therefore, halophytes can be used in saline-alkali land restoration, and are suitable to research the plant salt tolerance mechanism. More and more studies focus on the mechanism of plant salt tolerance from the molecular perspective, but few reports on *Nitraria tangutorum* have been published.

Genetic engineering is widely used in the study of plant salt tolerance and plays an important role in the study of its molecular mechanism (Wei et al., 2017). Recent studies have shown that *SOS2* is a key gene controlling plant salt resistance: *SlSOS2* in tomato increases salt tolerance (Baghour et al., 2019), overexpression of *PtSOS2* improves tolerance to salt stress in poplar (Yang et al., 2015) and the *MdSOS2* gene contributes to the salt tolerance of apple callus and improves the salt tolerance of transgenic Arabidopsis (Hu et al., 2012).

The germination rate of seeds under salt stress is related to the concerning plant’s salt tolerance. Salt stress can increase the seed coat’s permeability, and salt ions may damage the seed’s embryo, one of the main reasons for decreases in germination rate (Deng et al., 2014). In our study, overexpressing *NtSOS2* increases germination rate (Figure 5) under salt stress, indicating that ectopic expression of *NtSOS2* increases salt tolerance in *Arabidopsis*.

Salt stress restricts the growth and development of plants and may even cause plant death (Acosta-Motos et al., 2017). Here, we found that exogenous expression of *NtSOS2* improves salt stress tolerance in *Arabidopsis*, as it increases biomass and root growth and reduces leaf mortality (Figures 6, 7). These findings are analogous to and corroborate previous studies on *SOS2* gene activity (Wang et al., 2004).

The stress of adversity often leads to the changes of physiological state in plants.

Plants under salt stress often show the phenotype of leaf albinism and reduced chlorophyll (Taïbi et al., 2016). The content of MDA often reflects the degree of lipid peroxidation in the body and indirectly reflects the degree of cell damage (Jan et al., 2017). And also the improvement of antioxidant enzyme activity is an important factor to protect cells under stress (Farhangi-Abriz and Torabian, 2017). In this experiment, the transgenic Arabidopsis showed less chlorophyll loss, higher soluble protein and SOD accumulation under salt stress, and showed lower MDA accumulation (Figures 8, 11). All these indicated that the transgenic lines had better physiological state and stronger salt tolerance ability under salt stress, indicated that exogenous expression of SOS2 could improve salt tolerance of plants under stress.

Such studies have shown that *SOS2* plays an important role in maintaining a plant cell’s ion balance. In this study, exogenous expression of *NtSOS2* affects the expression of *AtSOS1*, *AtCAX1*, and *AtNHX1* genes, increasing it significantly in comparison to wild-type Arabidopsis (Figure 12). SOS2 can form a complex with SOS3 to assist the Na^+^/H^+^ antiporter SOS1, promoting the efflux of Na^+^ from plant cells to maintain a balance between ions inside and outside the cell, reducing ion toxicity (Ji et al., 2013). In non-halophytic plants such as *Arabidopsis*, the SOS2 complex activates the expression of SOS1 to enhance the salt tolerance (Quintero et al., 2002; Lin et al., 2009), and the same effect has been found in halophytic plants such as *Thellungiella salsuginea* (Oh et al., 2009, 2010). Similarly, the result observed in this experiment are similar to them (Figure 12A) which indicated that SOS pathway is conserved response to salt stress in plant. In addition, the SOS2/SOS3 complex can inhibit the high-affinity K^+^ transporter HKT1 to reduce the inward transport of Na^+^ and maintain Na^+^ homeostasis (Mahajan et al., 2008). Vacuoles can maintain the stability of cellular morphology and are important organelles in the process of plant stress resistance. Under salt stress, CAX1 can promote Ca^2+^ transport into the vacuole as well as outward H^+^ transport, while SOS2 can further stimulate this process (Cheng et al., 2004). In addition, SOS2 may interact with CBL10 to activate NHX1 and promote Na^+^ influx in vacuoles, maintaining their ion balance (Quan et al., 2007). It’s possible that the improved salt tolerance that observed in Arabidopsis overexpressing *NtSOS2* exogenously is due to *NtSOS2* similarly affecting the activity of ion transporters and maintaining the K^+^ and Na^+^ balance in plant cells under salt stress. Further studies will be required to confirm these hypotheses.

## Conclusion

In this study, a new gene *NtSOS2* was obtained from the halophyte *Nitraria tangutorum* and some functional verifications were done. Our findings suggest that *NtSOS2*, like its homologs from other plant species, stimulates salt tolerance. First, *NtSOS2* responds to salt stress in *Nitraria tangutorum* by increasing its expression. Second, overexpression of *NtSOS2* in *Arabidopsis* improves its salt tolerance and increases survival under salt stress. These results indicated that *NtSOS2* plays an important role in saline-alkali stress resistance, which is also of considerable significance to further understand *Nitraria tangutorum’s* mechanism of salt-tolerance. However, more research is needed to determine the molecular mechanism of *NtSOS2* function and to fully understand how exactly *Nitraria tangutorum* copes with salt stress. Such findings should eventually aid in the discovery of new methods to restore overtly saline soil.

## Data Availability Statement

The datasets presented in this study can be found in online repositories. The names of the repository/repositories and accession number(s) can be found in the article/Supplementary Material.

## Author Contributions

JC and TC contributed to the design of this research. LL, YLu, ZH, and JS carried out the statistical analysis. ZL, ML, JH, LZ, and YLi performed the experiments. LZ wrote sections of the manuscript. All authors contributed to manuscript revision and approved the submitted version.

## Conflict of Interest

The authors declare that the research was conducted in the absence of any commercial or financial relationships that could be construed as a potential conflict of interest.

## Publisher’s Note

All claims expressed in this article are solely those of the authors and do not necessarily represent those of their affiliated organizations, or those of the publisher, the editors and the reviewers. Any product that may be evaluated in this article, or claim that may be made by its manufacturer, is not guaranteed or endorsed by the publisher.

## Supplementary Material

Supplementary Figure 1 Agarose gel electrophoresis showing *NtSOS2* PCR. The first lanes is 2,000 DNA marker and the second to fifth lanes are *NtSOS2* genes from four separate seedings.

Supplementary Figure 2 Protein domains within the NtSOS2 protein as shown by NCBI CDD (https://www.ncbi.nlm.nih.gov/cdd).

Supplementary Figure 3 Semi-quantification of *NtSOS2* in transgenic Arabidopsis.

Supplementary Figure 4 Prediction of NtSOS2 subcellular localization by ProtComp9.0 (http://linux1.softberry.com/berry.phtml).

Supplementary Table 1 Primers for Quantitative RT-PCR.

Supplementary Table 2 Primers for Subcellular localization. The underlined bases indicated the location of the restriction sites.

Supplementary Table 3 Primers for constructing overexpression gene vector. The underlined bases indicated the location of the restriction sites.

Supplementary Table 4 *SOS2* gene and its sequence number in different species used in DNAMAN.

Supplementary Table 5 The CIPK genes used to construct phylogenetic tree in Arabidopsis and rice.

Supplementary Table 6 Primers for isolation of *NtSOS2* fragment.

## Abbreviations

SOS: Salt Overly Sensitive
CIPKs: Calcineurin B-like proteins-Interacting protein kinases
NAF: Asn-Ala-Phe
FISL: Phe-Ile-Ser-Leu
ORF: Open Reading Frame
CDS: Coding sequence
Cdna: Complementary DNA
qPCR: Quantitative real-time PCR
OE: Overexpress
HKT1: high-affinity K^+^ transporter 1 gene
NHX1: Na^+^/H^+^ exchange 1 gene
SOS1: Salt Overly Sensitive 1 gene
CAX1: Ca^2+^/H^+^ Cation Exchanger 1 gene.
