# Supplementary material for: Overexpression of NtSOS2 From Halophyte Plant N. tangutorum Enhances Tolerance to Salt Stress in Arabidopsis
Source: Front Plant Sci. 2021 Sep 6;12:716855. doi: 10.3389/fpls.2021.716855 (PMC8450600; doi:10.3389/fpls.2021.716855)
Supplement: Supplementary Figure 1 — Agarose gel electrophoresis showing NtSOS2 PCR. The first lanes is 2,000 DNA marker and the second to fifth lanes are NtSOS2 genes from four separate seedings. [file Data_Sheet_1.docx]

**
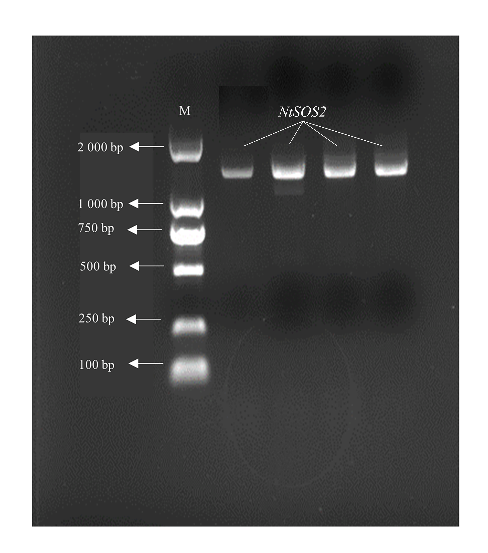
**

**Fig. S1** agarose gel electrophoresis showing *NtSOS2* PCR. The first lanes is 2 000 DNA marker and the second to fifth lanes are *NtSOS2* genes from four separate seedings.


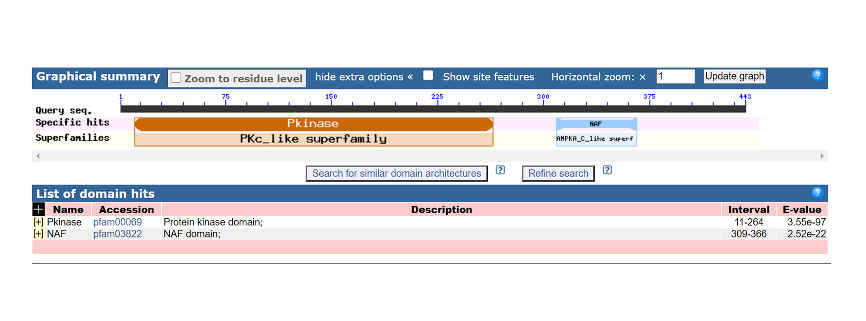


**Fig. S2** Protein domains within the NtSOS2 protein as shown by NCBI CDD (<https://www.ncbi.nlm.nih.gov/cdd>).


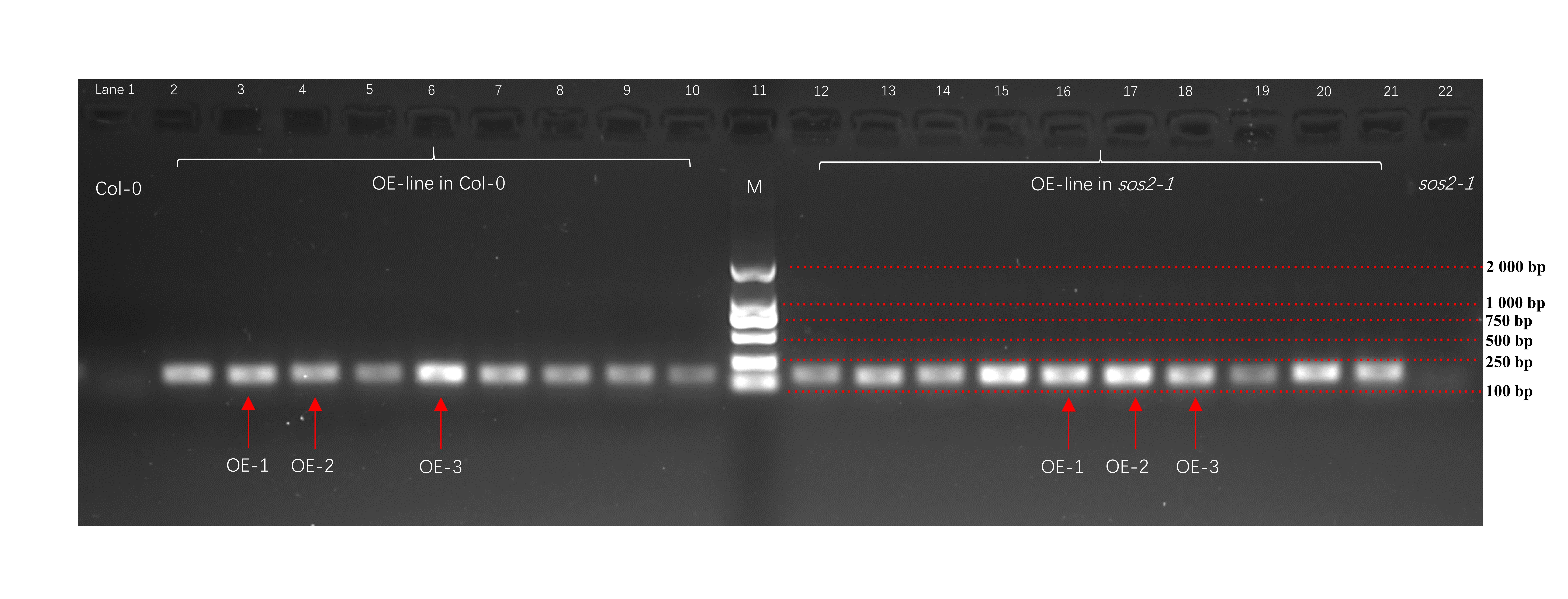


**Fig. S3.** Semi-quantification of *NtSOS2* in transgenic Arabidopsis. The overexpressed lines used in the article in the wild type and mutant *sos2-1* are marked with red arrows respectively.

**Fig.S4**

| Pentamers | Integral |
| --- | --- |
| 0 | 0 |
| 0.06 | 9.65 |
| 0.23 | 0 |
| 1.58 | 0 |
| 2.4 | 0.16 |
| 0 | 0 |
| 0 | 0.03 |
| 0.39 | 0 |
| 0.13 | 0.15 |
| 0 | 0 |

**Fig. S4.** Prediction of NtSOS2 subcellular localization by ProtComp9.0 (<http://linux1.softberry.com/berry.phtml>).
